# Reply to Lang et al.: The use of DEMs versus DSMs in viewshed analysis

**DOI:** 10.1073/pnas.2409845121

**Published:** 2024-08-05

**Authors:** Wei Guo, Leonie Wenz, Maximilian Auffhammer

**Affiliations:** ^a^Euro-Mediterranean Center on Climate Change Foundation, Leece 73100, Italy; ^b^Resources for the Future - Euro-Mediterranean Center on Climate Change European Institute on Economics and the Environment, Centro Euro-Mediterraneo sui Cambiamenti Climatici, Milan 20144, Italy; ^c^Department of Complexity Science, Potsdam Institute for Climate Impact Research, Potsdam 14412, Germany; ^d^Mercator Research Institute on Global Commons and Climate Change, Berlin 10829, Germany; ^e^Department of Agricultural and Resource Economics, University of California, Berkeley, CA 94720; ^f^National Bureau of Economic Research, Cambridge, MA 02138-5398

We appreciate the comments of Lang et al. (1). They are concerned that the ground elevation data we (2) employed are not sufficient for viewshed analysis of wind turbines. They argue that employing a Digital Surface Model (DSM), which accounts for trees and buildings, is a superior approach. In contrast, our usage of the Digital Elevation Model (DEM) could result in measurement error. We do not argue that better data on what is “in the way between a home and a wind turbine” will result in lower measurement error. We explicitly address this in our robustness checks comparing areas with different building heights (*SI Appendix*, Table S3). We chose a DEM over a DSM for three primary reasons:

First off, the geographic scale of our paper is national. At the time we conducted the analysis, we could not find a DSM at the national scale. Further, we would require one, which is updated at regular intervals, to account for changes in the stock of buildings and trees going back to 1997—the beginning of our data. As far as we know, such a product is not available today either.

Second, several regions use elevation models that represent bare terrain only for wind farm planning (3–5). Approaches such as zone of theoretical visibility or visual influence employing bare terrain models for landform screening are widely used in wind project assessments (6, 7). Scotland’s Natural Agency (4) notes that: “*For most projects these datasets (DSM) do not make a significant difference to the pattern of visibility and they tend to be quite expensive… …therefore, their use should be limited to specific projects and viewpoints where the benefits will be notable […]*”.

Further, current large-scale DSMs generally suffer from significant vertical elevation errors (8). While both models are produced using aerial radar and with photogrammetry methods (9), DEMs are typically cross-validated using ground surveys (10). Moreover, DSMs are more prone to errors due to the inclusion of temporary objects and seasonal variations in vegetation (11).

Lang et al. (1) show that for Cuyahoga and Hardin counties in Ohio a DEM based on a different (Ohio Geographically Referenced Information Program) dataset misclassifies a significant share of properties as being in the viewshed of a wind turbine. We cannot assess whether the magnitude of this issue is the same nationally or for the data we used. We also cannot conjecture how this would influence the point estimates in the hedonic model. We would be excited to see a paper that uses a DSM for a larger area and time period and compares the hedonic for the two models. We conjecture that the estimates would rise. In our paper, we have been clear about what we are estimating (landscape-based viewshed) and do not intend to evoke the impression that we are estimating what a DSM can do via a DEM. We also note that one of the more interesting findings in our paper is that the disamenity value diminishes in time and space, which should be robust to this misclassification concern.
